# Material extrusion additive manufacturing of dense pastes consisting of macroscopic particles

**DOI:** 10.1557/s43579-022-00209-1

**Published:** 2022-08-03

**Authors:** Alexandra Marnot, Alexandra Dobbs, Blair Brettmann

**Affiliations:** 1 Chemical and Biomolecular Engineering, Georgia Institute of Technology, Atlanta, USA; 2 Chemical and Biomolecular Engineering, Materials Science and Engineering, Georgia Institute of Technology, Atlanta, USA

**Keywords:** 3D printing, Additive manufacturing, Composite, Particulate, Paste, Mixture

## Abstract

Additive manufacturing of dense pastes, those with greater than 50 vol% particles, via material extrusion direct ink write is a promising method to produce customized structures for high-performance materials, such as energetic materials and pharmaceuticals, as well as to enable the use of waste or other locally available particles. However, the high volume fraction and the large sizes of the particles for these applications lead to significant challenges in developing inks and processing methods to prepare quality parts. In this prospective, we analyze challenges in managing particle characteristics, stabilizing the suspensions, mixing the particles and binder, and 3D printing the pastes.

**Graphical abstract:**

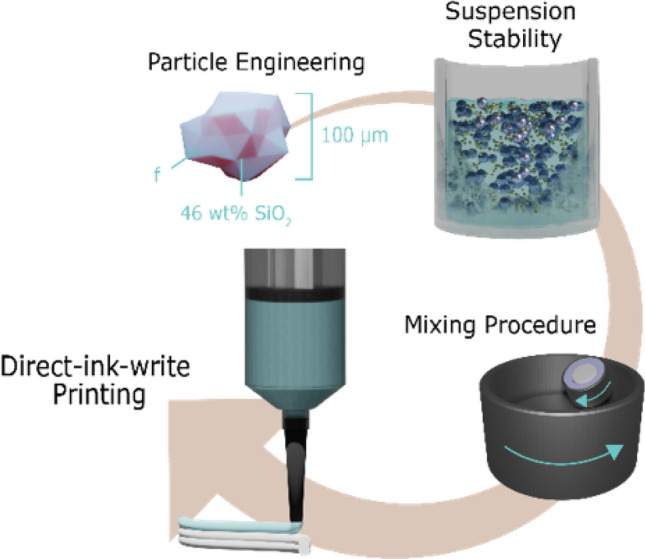

## Introduction

**Figure 1:**
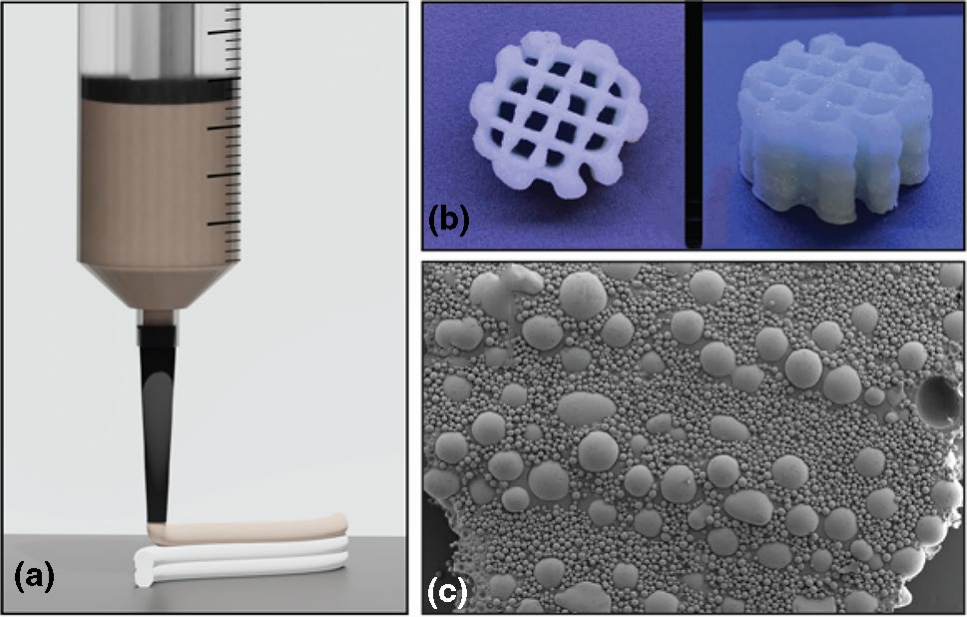
(a) Illustration of the DIW process with a syringe depositing the dense paste ink layer by layer, (b) Top and side view of a 10-layer print from 65 vol% soda-lime glass microspheres suspended in a polymeric binder, (c) SEM image of a cross section of a 65 vol% soda-lime glass microspheres printed line.

Additive manufacturing has come to prominence in making customized parts and unique geometries for a wide variety of applications including medical implants,^[1,2]^ energetic materials,^[3,4]^ pharmaceuticals,^[5,6]^ electronic devices^[7,8]^, and more. Material extrusion additive manufacturing, especially at room temperature, where it is often called direct ink writing (DIW) or robocasting, is particularly promising for customized formulations and where there are tight safety and control considerations in formulation and/or in processing. This is because the components are isolated from the environment in the syringe and there is no heat applied. In DIW, an ink is continuously extruded from a nozzle as a filament and deposited layer by layer (Fig. 1). A major advantage of DIW is the flexibility and customizability of the ink; gels,^[9]^ pastes,^[10,11]^ liquid crystalline elastomers^[12]^, and resins^[13]^ have all demonstrated success as long as the rheology of the ink can be adapted to flow in the nozzle and maintain shape as a filament. With this wide array of materials that can be printed via DIW, both understanding how the formulation affects printability and developing design rules for the customized inks are particularly important. In this prospective, we hone in on one key regime for DIW inks: macroscopic particles at high particle loadings.

High particle loading suspensions, here defined as > 50 vol% particles and also called “dense pastes,” consist of particles suspended in a binder, typically a polymer solution or monomer/pre-polymer that is later cured. Dense pastes are of particular interest in applications where the particle provides the functionality and the polymer is only a binder, so maximizing particle content is valuable.^[3,4]^ In some applications of dense pastes, the presence of the binder in the final product can be an inconvenience to the end application. To this extent, significant work has been done to utilize material extrusion/DIW in conjunction with binder-free materials. For instance, in regenerative medicine, the presence of organic binders in 3D printed bone cements presents cytotoxicity concerns, while for 3D printed thermoelectric materials in the energy sector, binders can decrease the electrical conductivity performance.^[14–16]^ Despite this, for the successful extrusion of binder-free pastes, a carrier liquid is still required. For the scope of this prospective, we focus on the challenges associated with processing of binder-containing formulations. Dense pastes cover a range of particle loadings, from the concentrated suspension regime (> 40 vol% for monodisperse spherical particles) to the slurry regime (~ 65 vol% or near the maximum packing fraction) to the wet granular regime (> 65 vol%). Note that there is not a single definition and cutoff for concentrated suspension vs. slurry and we use the terms as defined here, but literature terminology may differ, especially from one application space to another. The cutoffs for the regimes will also differ for different particle types (spherical vs. anisotropic) and different particle size distributions (e.g., monomodal vs. bimodal). Thus, there is a rich parameter space for designing AM processes for dense pastes, including not just the vol% particles, but also the particle surfaces (roughness^[17,18]^ and chemistry^[19]^), binder choice,^[3]^ particle shape^[20]^, and whether a mixture of particles is employed.^[10]^ One of the most important parameters, however, is the size of the particles. We can consider them in two regimes: macroscopic particle size (> 1 µm) and colloidal particle size (1 nm to 1 µm). When considering ink design for DIW, some formulation and processing considerations will be the same for both regimes, while others will not. It is important to consider the similarities and differences between the two regimes when designing a printing process for dense pastes, especially due to the large volume of particles in the formulation.

Macroscopic particles play a particularly important role in 3D printing dense pastes for growing application areas. Recently, AM for building construction garnered momentum with NASA, in light of the Artemis 2024 mission and the Moon to Mars Planetary Autonomous Construction Technologies. With the goal of establishing habitats off-world, in situ resource utilization (ISRU) presents an attractive solution to offset the tradeoff between the need for high-performing materials and the high costs of space launches. While thus far, extraterrestrial materials considered for ISRU include polar ice water, solar radiation, and regolith, for the context of this article, the use of the latter resource is the most applicable. Example particles in this case are Lunar and Martian regolith, composed primarily of metal oxides with particle sizes *D*_50_ of 70–150 µm.^[21,22]^ Current investigations into 3D printing of Lunar and Martian regolith using DIW have attempted to replicate traditional concrete, which cures via hydration between water and Portland cement followed by excess water evaporation, and demonstrated the applicability of solvent-induced reaction and solvent evaporation as solidification mechanisms.^[23]^ Another appealing application of printing macroscopic particles is re-/upcycling of waste materials, including fly ash from coal plants.^[24,25]^ In both of these applications, inclusion of large volumes of particles is important to maximize the amount of local or waste materials used and to minimize the need for additional, virgin polymer binder. Another area where high volumes of particles are desirable is when the performance demands are high and the particles provide all functionality. Energetic materials, such as propellants and explosives, are examples of this where macroscopic organic crystals, aluminum and other energetic materials are required at very high proportions (> 80 vol%), but additive manufacturing is attractive for designing higher performance structures.^[3,4,26,27]^ Similarly, 3D printing pharmaceutical tablets can open the door to enhanced designs such as polypills, or tablets containing multiple different drugs, and lattice structures for improved dissolution.^[5,6]^ However, the particle sizes of drugs and common excipients, such as disintegration aids, are in the macroscale range and the tablets often require high drug particle loadings to ensure sufficient dosage and release properties.

Despite the potential impact of using material extrusion to 3D print dense pastes of macroscopic particles, a number of challenges hold back this technology. In this prospective, we discuss the impacts of particle characteristics, including the particle size distribution, shape, and roughness. We also consider the meaning of a “stable” dense paste, potential sources of destabilization, and approaches to improve stability. Finally, we examine the key processing technologies, both for mixing and printing, and the unique considerations for macroscopic particles and dense pastes. When examining these different aspects and challenges in DIW dense pastes, we focus on the role of the particles themselves and do not significantly address the non-particle components such as the binder, surfactants, etc. These components would also play an important role in designing a dense paste formulation, but are outside the scope of this prospective. We finish with an outlook for the key research questions remaining to understand and improve material extrusion additive manufacturing of dense pastes of macroparticles.

## Challenges in AM dense pastes of macroparticles

### Particle characteristics

The size and shape of particles plays a significant role in the flow and packing behavior of highly concentrated systems. This is important across many of the processes in DIW of dense pastes, from the flow during printing to the stability of the suspensions. For example, bimodal distributions of particles near the ideal packing ratio for the particle sizes have been shown to lead to fluids with lower tan-delta and improved flowability.^[10]^ And monomodal distributions of small particles as well as elongated particles, such as needles, have been shown to have significantly higher viscosities due to the high surface area for interactions with other particles.^[28,29]^ Thorough characterization of particles, especially their size distributions and shapes, is essential to designing a formulation for DIW, but different particles require different characterization techniques and careful consideration of how to best characterize is required.

**Figure 2:**
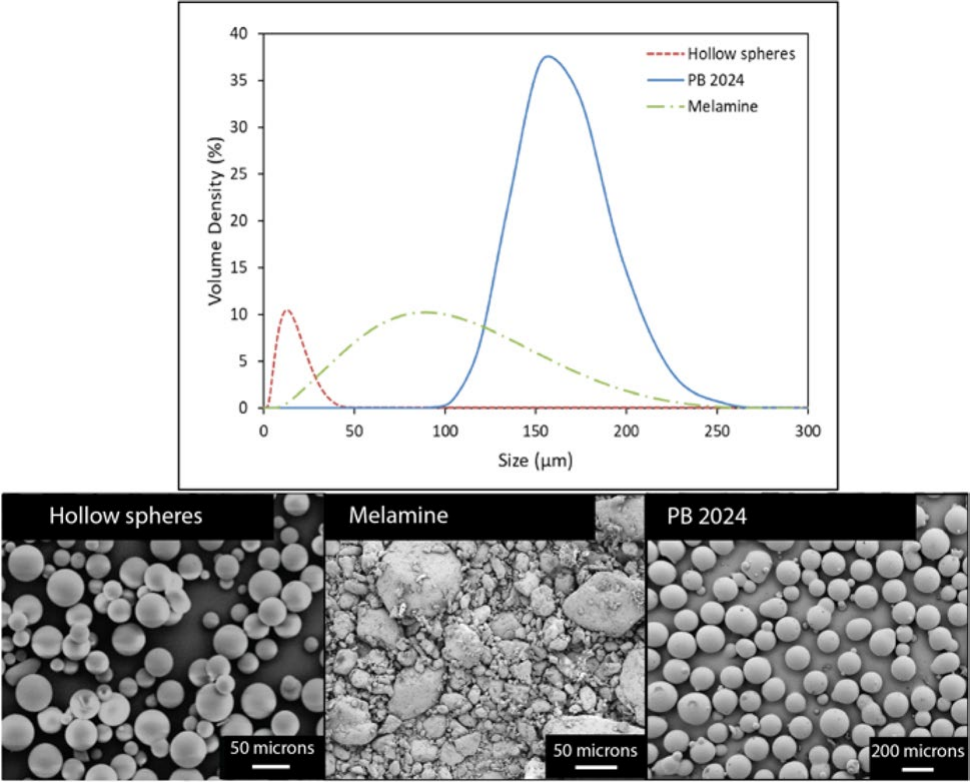
Laser diffraction data and corresponding SEM images of three macroscale particle types: hollow spheres (dashed red curve), melamine (dashed green curve), and PB 2024 (blue curve).

One element of consideration is the size range of the particles, while another is whether the particles are synthesized in a controlled environment, such as pharmaceutical crystals, or sourced from natural products or waste streams, such as soil. For particles in the colloidal regime, dynamic light scattering (DLS) gives good resolution for the particle size distribution. DLS, however, is not suited for the measurement of particles larger than 10 μm and must be conducted in the dilute range, imposing precise particle sampling and dispersion procedures.^[30,31]^ On the other hand, laser diffraction (LD) is recommended for analysis of particle sizes between 10 μm to several millimeters and can be utilized to measure dense suspensions. Laser diffraction data and corresponding SEM images of example powders are shown in Fig. 2. A weakness with laser diffraction is that it cannot distinguish the size distribution of smaller particles.^[32–34]^ This poses characterization difficulties for multimodal suspensions with particles that span a wide range of sizes and usually results in a certain margin of error. An additional factor therefore often needs to be included, in which particles are initially sorted and then appropriately characterized with the corresponding technique. Several techniques exist to automate the sorting process, such as hydrodynamic sorting or sensor-activated flow cytometry, but they are often expensive to implement, require lower particle concentrations than 50 vol%, or are time-consuming.^[35]^ For particles used in the space exploration, energetics, and re/upcycling of industrial waste, sorting is mainly achieved through means like automated sieve shakers or industrial conveyor belt sieve sorters. Addition of the initial sorting step can introduce further errors, as standard mesh sieves can trap irregularly shaped particles with rough surfaces and skew the characterization of the particle size distribution.

One example of a multimodal suspension in which multiple techniques need to be employed for a detailed characterization of the particle size distribution is extraterrestrial regolith for in situ resource utilization in space exploration efforts.^[36–38]^ Current work on simulated regolith particles has shown that the particles are not inherently suitable for processing, and thus must often be sieved or milled to achieve a desired particle size distribution that ensures suitable flow, shape retention, and weathering properties. Here, the manual alteration of the native particle, such as handling and milling, contributes to changes on the particle surface, increasing roughness^[39]^ and introducing contaminants.^[40]^ These surface changes affect the particle dynamics and can cause discontinuous processing and alter the end mechanical properties.^[17,18]^ Characterization of the particle size for these irregularly shaped particles through LD can also become difficult (see broad melamine peak in Fig. 2), as diffraction measurements relying on the Fraunhofer and Mie theories assume a spherical particle shape. Furthermore, sieving of irregularly shaped particles such as those found in simulated extraterrestrial regolith may inadvertently introduce a bias in particle shape when compared to the bulk sample.^[41]^

Due to contamination concerns or hazards in handling of some particles, it is not always possible to sieve a bulk sample to either assess or obtain a specific size distribution. In these cases, image analysis is sometimes used for the particle size and morphology. In the most simple case, optical and scanning electron microscopy (SEM, Fig. 2) is done on a sample of many particles, image analysis is performed to measure the particle sizes of a representative number of particles, and averages are calculated. This can be a good estimation if enough particles are measured and there is no bias in selection of which to measure. The other case for image analysis is typically applied to process control, especially for crystallized particles, where characterization of the size and shape is crucial for achieving final product properties, such as dissolution profiles and stable shelf-life for pharmaceutical crystals, and it is desirable to have real-time monitoring of crystal size and shape during crystallization.^[42,43]^ The crystal shape growth can be predicted by models based on image analysis during crystallization, but the technique becomes quantitatively difficult for dense drug suspensions.^[44]^ Traditionally, the focus of particle engineering efforts in drug crystallization revolves around fine-tuning parameters such as concentration, solubility, and nucleation kinetics to achieve a resulting particle morphology that meets required end properties.^[45,46]^ In both these approaches, optical image analysis is often sufficient for macroscale particles, while SEM or TEM is necessary for colloidal particles. This makes it easier to characterize the particle size of macroscale particles via image analysis and enables in-line characterization during processing as is seen with the pharmaceutical crystallization. Despite this, work remains to be done in bridging these same particle size parameters and in characterizing the morphology requirements for flow, shape retention, and particle spatial distribution in DIW 3D printing.

So far, we have focused primarily on the inherent particle size characteristics, but there is significant work in particle engineering throughout many industries, including those interested in additive manufacturing of dense pastes of macroscale particles. Some particle engineering approaches include controlling the shape of the particles, especially aiming for spherical particles, which lead to more continuous flow and denser prints.^[45]^ One approach to preparing spherical particles is to precipitate or crystallize from water-in-oil emulsions, which consists of forming spherical water droplets with the precipitating molecule localized to the water phase and using evaporation to crystallize into spherical agglomerates.^[47,48]^ Another key focus of particle engineering is tailoring the surface of the particles. The flowability of powders depends on the surface characteristics of the particles, including the chemistry and roughness, and this is expected to be the case for dense pastes as well due to the high contact surface area. Some approaches to improve flowability via surface engineering include dry coating macroscale particles with nanoparticles and silane functionalization.^[49]^ Despite the extensive prior work on surface modification for improving flow of powders, further study is needed for how the fluid layer in dense pastes impacts these approaches and to design enhanced particles that improve flow during additive manufacturing.

### Stability

A suspension’s stability has critical implications on the quality of the printed product*.* Stability is a term used in this context to reference a suspensions ability to maintain, or re-attain after a disturbance, a homogeneous particle microstructure. Suspensions should ideally preserve a homogeneous particle distribution throughout processing, printing, and storing, as heterogeneities may lead to inconsistent line printing and/or nozzle clogging. Dense pastes form heterogeneities when applied forces, such as shear forces in the nozzle and gravity, induce particle migration, size segregation, and/or binder separation.

**Figure 3:**
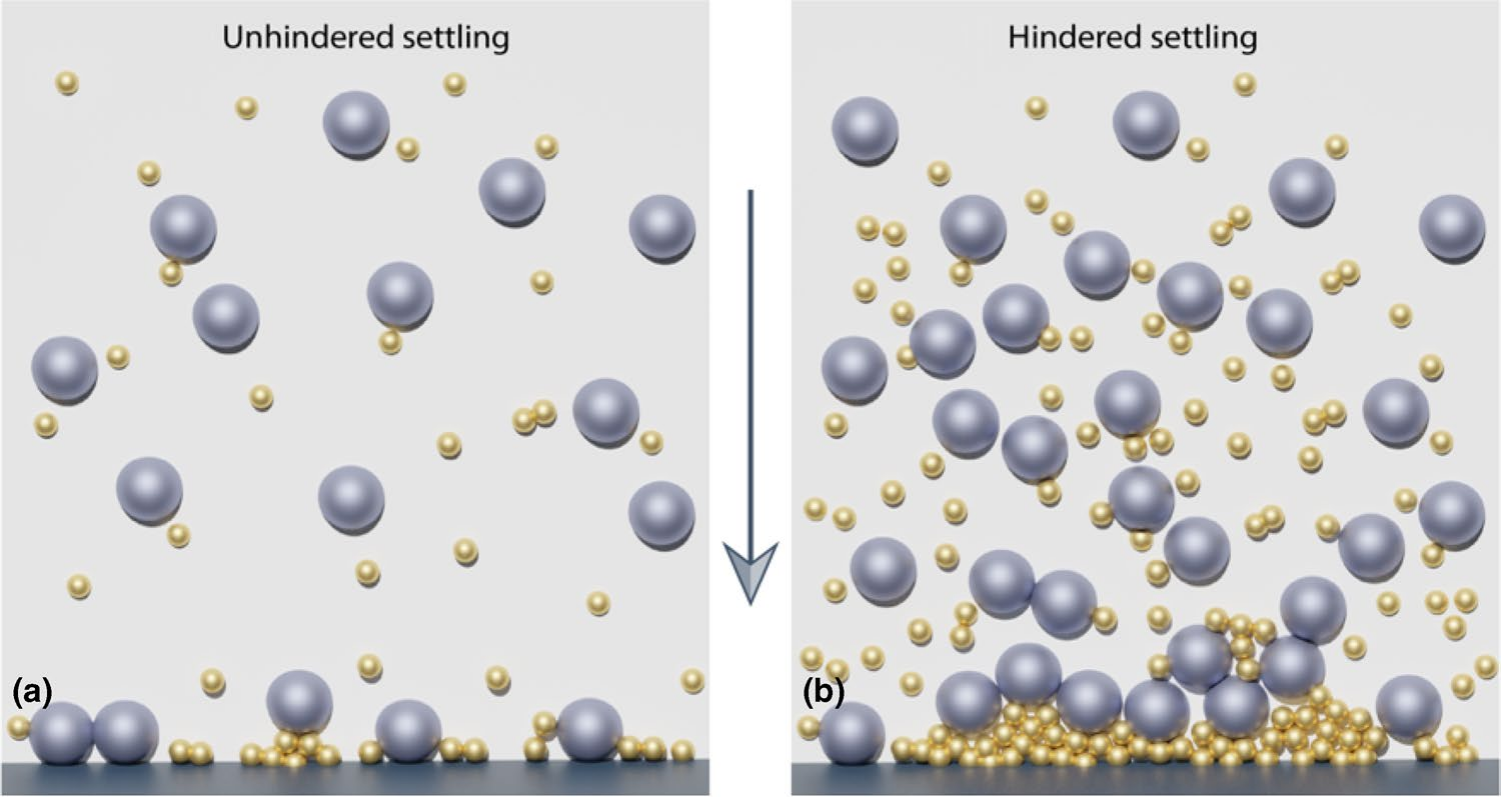
(a) Free settling/unhindered settling of a bimodal distribution of particles. (b) Hindered settling of the same particles at a higher particle concentration. Increased and frequent particle interactions during the fall result in small particle-rich regions at the bottom.

Settling and binder leaching are two descriptions of heterogeneity formation in dense pastes, where settling is sedimentation of particles due to gravity and binder leaching is the separation of the binder and solid phase due to insufficient capillary forces. These can occur concurrently and are primarily distinguished by whether the particles or the fluid is doing more of the movement. Hindered settling (Fig. 3) occurs in dense pastes as the many particle–particle interactions lead to distorted velocity fields and thus longer settling times. The Richardson and Zaki model describes settling velocities as a function of packing fraction, particle and binder densities, and particle size, and it is often used due to its simplicity.^[50–52]^ Alternative models that are more accurate for solids concentrations near the maximum packing fraction and solids that are polydisperse/polydense or irregularly shaped have also been presented.^[53,54]^ Settling can impact rheological measurements for concentrated suspensions and convolute interpretations of shear thickening behavior.^[55]^ If the suspension contains particle types with sufficiently different settling velocities, the slower particles begin to form a layer on top, called creaming. Compression settling is unlikely in DIW but may occur if the overhead weight of the material in an extrusion barrel or syringe becomes too large.^[50]^

Binder leaching (also referred to in the literature as bleeding or weeping) is another description of binder–particle separation and can occur through many mechanisms including when fluid travels to the surface as particles settle and when the capillary pressure is insufficient to maintain a binder meniscus at the binder gas interface. The capillary pressure is influenced by the binder surface tension, particle wettability, and the distance between particles.^[56,57]^ Binder separation may also occur under applied shear in capillary flows as particles migrate away from the wall and form a dense particle mat across the die in the extruder, leading to filtration and extrusion of only the binder until the mat is broken up and suspension flow begins again.^[58]^ Fundamental studies on suspension behavior provide excellent insight into the physics of particle and binder interactions. However, the relationships between formulation factors and stability in dense pastes of macroparticles are still emerging, and implication on print quality is a rich area for discovery.

The most common approaches for achieving stability in dense pastes of macroparticles are to manage binder surface tension, binder/particle interfacial tension, viscosity, and density, or to manage particle volume fraction, size/shape and their distributions, surface roughness, and density. Slower settling velocities can be achieved by increasing the particle volume fraction, matching the fluid and particle density, or by operating at lower temperatures.^[55]^ At high solids concentrations, the mechanism of settling and size segregation may depend on the density difference between the particle and fluid. Snabre et al. suggest a jamming transition (where a layer of particles spanning the horizontal pane of the vessel forms and is maintained during settling) may occur when the density fluctuations are not large enough to melt the percolating structure. They also report less size segregation of small and large particles (creaming) from the jammed structure.^[59]^ Binder leaching can be addressed by using binders that fully wet the particle surface and have sufficient capillary forces to form strong binder-particle bridges, and by minimizing interparticle distance. Vandevivere et al. found that binders that wet the solid, have low surface tensions, and have high viscosities form durable structures.^[60]^ Ho et al. found that increased binder wetting promoted interparticle contacts and reduced void space in the final product.^[61]^ This binder leaching can be reduced by introducing small particles to sufficiently fill interstitial voids and decrease interparticle distances. Binder wettability can be determined using methods proposed by Owens and Wendt combined with contact angle (sessile drop method) and surface tension measurements (pendant drop, or Wilhelmy plate methods).^[60–62]^ Balancing formulation factors is an ongoing challenge for DIW AM of dense suspensions of macroparticles, as properties that promote suspension stability and shape retention often negatively impact printability factors such as the pressures required to flow.

In general, issues such as settling, and binder leaching are less common for colloids since small, light particles have long settling times and Brownian motion that dominates gravitational force. Settling may occur in colloids if large agglomerates form and the suspension is not density matched. Heterogeneity formation in colloids is typically a concern of particle coalescence, the exothermic contact of particles to reduce total surface area, and, therefore, surface energy. Smaller particles have more surface area and as a result are less stable due to Van der Waals attractive forces that result in agglomeration. Stabilization can be achieved by energetically inhibiting particle–particle coalescence via electrostatic repulsions or steric hindrance.^[63,64]^ This can be done by adding dispersants/stabilizers such as surfactants, polymers, ligands, and dopants, or modifying zeta potential (solvent quality, pH, surface forces). Particle loading and electrosteric forces can be manipulated to change the particle microstructure from a liquid to a gel or glass like state, which may form spanning structures when printed.^[65]^ Microstructures are disordered with gel like states being ergodic and having sufficient connectivity to form a coherent mass; while glass like states are non-ergodic and fully connected.^[66]^ Glass like microstructures can take on different forms depending upon the forces at play,^[67]^ and can and cause changes in suspension viscosity, modulus, and yielding behavior; which may negatively impact printability and processing.^[68,69]^ Highly positive or negative zeta potentials may reduce or promote agglomeration in macroparticles due to attractive or repulsive forces in suspensions;^[70]^ however, electrostatic forces are unlikely to have a significant impact on settling behavior when the particles are greater than 1 μm and no longer in the colloidal regime. Using bimodal distributions may fill voids in the printed structure, but induces other challenges such as particle agglomeration and nozzle clogging.^[71]^ Colloidal particles (cornstarch) have also been used as a shear thickener for macroparticle suspensions.^[55]^ Additionally, small amounts of a wetting/non-wetting fluid can be added to particle suspensions of macro- and nano-sized particles to improve stability and settling.^[72]^ While some approaches toward improving stability in dense suspensions are similar for colloidal and macroparticles, much of the focus for colloidal particles is on controlling particle interactions to prevent agglomeration or control gelation, while for macroparticles, it is on managing binder leakage and settling.

**Figure 4:**
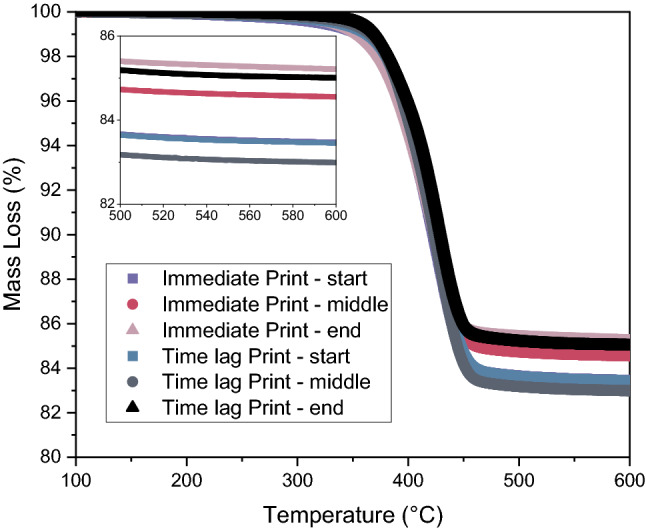
Mass loss vs. Temperature for prints with ink printed immediately after mixing and after 24 h (time lag). Three samples were analyzed for the print: start of the printing time, middle of printing time, and end of printing time.

Another element of stability is the aging of the inks. This can include the sedimentation and binder leaching described in this section, but it can also include factors such as solvent evaporation, premature crosslinking and many other mechanisms frequently seen in suspension formulation. In dense pastes (defined here as > 50 vol% particles), this phenomenon is typically not reported in the literature, though at lower solids, researchers have seen aging lead to higher storage modulus in rheological testing and concurrent improved printability at longer aging times. For 65 vol% glass particles in 2:1 vol:vol PEDGA:2-ethylhexylacrylate, we examined whether there was a difference in binder/particle segregation if printing occurred immediately after mixing or after 24 h. Figure 4 shows the mass loss as a function of temperature measured through thermogravimetric analysis for samples taken from the beginning, middle and end of the print. The mass loss corresponds to the fraction of binder in the material. At the start of the print, the immediate vs. aged samples show no difference in fraction of binder (purple and blue curves lie on top of one another). Middle of the print samples shows that, at immediate times, there is less binder than at the start of the print, whereas after aging, the middle print sample lines up well with the start of print sample. For both immediate and aged samples, there is less binder at the end of the print. This indicates that aging may play some role in phase separation of binder from particles at these high solids, but that analysis of more systems is necessary to conclusively understand the aging process.

### Mixing and sample preparation

High solids suspensions are difficult to mix due to their high viscosities that require high shear to achieve homogeneity. Blade impellors are typically undesirable as they may not be able to generate shears high enough to thoroughly mix viscous suspensions, and particle collisions with the blade may either cause particles to fracture or abrase the blade, which could lead to contamination of the suspension.^[73]^ Additionally, high shears are localized at the blade tips and may create hot spots within the mixture, potentially leading to combustion for primary explosives. Viscous material deposited on the blades is also difficult to clean and results in material losses in addition to presenting safety concerns in the case of energetic materials.^[73–75]^ Bladeless methods such as dual-axis centrifugal mixers (also called dual asymmetric centrifuge (DAC), rotation revolution, high speed/blade free planetary mixer, planetary centrifugal) and resonant acoustic mixers (RAM) are common for high solids suspensions. In addition to being bladeless, these mixers are easy to clean, allow for the use of different vessels that are sealed (which may help reduce evaporation, provide for safety, etc.), and result in more uniform mixing than with blades. Other methods used for mixing high solids suspensions include twin screw or shear roll (such as two/three-roll milling) extruders, twin-type kneader, and batch internal mixing.^[20]^ Shear roll milling has a higher energy input than twin screw extrusion; however, it requires additional processing, such as scraping and pre-milling, and should be avoided when volatile solvents are present in the formulation.^[76,77]^ The appropriate mixing method for high solid suspensions depends on formulation factors such as viscosity, particle size, and solvent.

Both DAC and RAM have been widely used to achieve good mixing in highly filled suspensions. In RAM mixers, the vessel is vibrated vertically at a set acceleration of up to 100 G, and mixing occurs due to Faraday instabilities at the boundary between materials with different densities. This results in low shear mixing that is uniform throughout the vessel. DAC mixers generate dual centrifugal forces from rotation around a central axis and revolution about an axis that is tilted, with respect to the revolution plane. DAC mixers generate high shears throughout the vessel and can de-foam the material to produce mixtures free of air bubbles. For both RAM and DAC, higher mixing speeds (acceleration for RAM, and rpm for DAC) result in lower mixing times.^[78,79]^ Claydon et al. discussed the influence of wall slip behavior for RAM mixers and showed that higher surface energy vessels may promote mixing. They also report that mixer intensity and mixing behavior varied between different mixers and suggest using calibration for mixing studies.^[78]^ RAM mixers may generate lower shears (and thus lower heat rise) than DAC mixers and be more appropriate for irreversibly shear thickening suspensions; however, the lower shears produced by RAM mixers may not be effective at breaking up particle agglomerates and thus require pre-mixing.^[73]^ Dense suspensions of colloids are prone to irreversible shear thickening, and Bird and Ravindra showed improved results with the use of a RAM mixer (LabRAM) as opposed to a DAC mixer (FlackTeck) for colloidal suspensions, as the DAC mixer was too aggressive and always produced shear-thickened suspensions.^[77]^ Although the driving forces and fluid mechanics of the two systems are very different, studies directly comparing DAC and RAM for dispersing dense pastes are sparse and further work is needed to develop guidelines for when each technique should be used in mixing pastes for additive manufacturing.

Following mixing, the degree of homogeneity is important to characterize as it may have implications on flow behavior and effective packing fraction, when the binder becomes trapped in agglomerates of poorly mixed suspensions.^[80]^ With a precise definition, dense pastes are never truly homogeneous, as they include the particle phase and the binder phase, so there will always be heterogeneities on the length scale of the particles. However, a practical definition of homogeneity can be defined based on larger length scales and what length scale of particle agglomeration and binder leakage leads to flow instabilities, defects in printed parts, and other negative outcomes. One useful definition in DIW is considering a dense paste “homogeneous” if particle agglomerates are less than 1/10 of the nozzle size, which is a typical rule of thumb to prevent nozzle clogging. Another useful definition is a sufficiently low level of binder leakage to prevent a binder-rich layer on the outside of a printed line, a phenomenon that was seen for some formulations in our previous publication.^[10]^ When designing a dense paste for DIW printing, the definition of homogeneous should be clarified at the start of the project.

**Figure 5:**
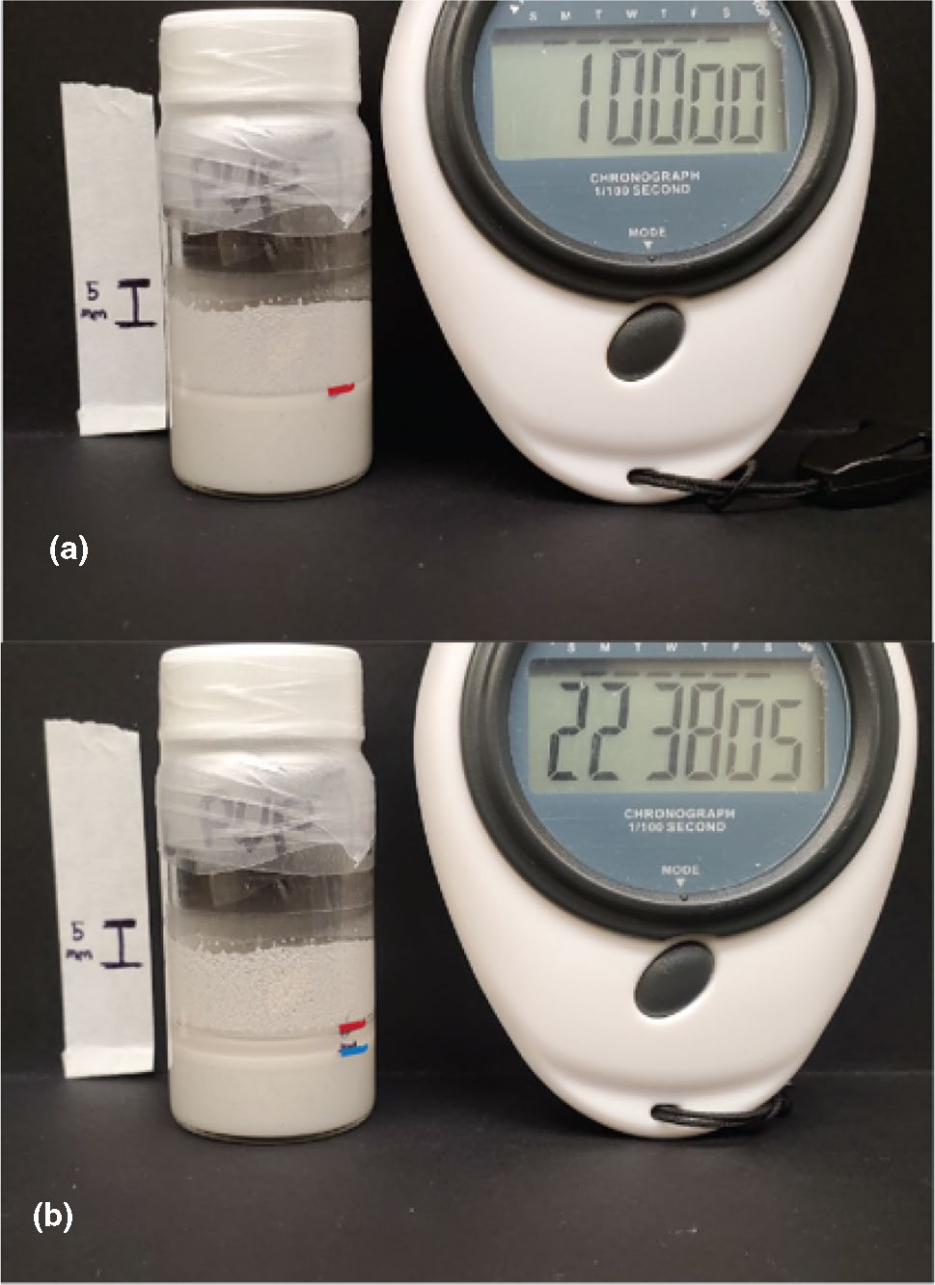
Visual settling tests showing (a) no separation at 1 h, (b) separation of large and small particles at approximately 23 h. White above the red line is residue on the vial wall and scale bar is 5 mm.

There are several qualitative and quantitative ways to measure how well particles are mixed with other particles and the binder and thus ensure mixing procedures are effective at producing homogeneous particle suspensions. Rueda et al. provide an extensive review of methods to determine dispersion including light scattering, X-ray diffraction, energy-dispersive x-ray (EDX), SEM X-ray mapping (SEM–EDX), backscattered electron (BSE) imaging, tomography coupled with TEM, and mixing torque rheology. They emphasize that optical results should be confirmed with rheology, image analysis (e.g., ImageJ software), multifractal studies, or statistics to ensure images represent the bulk dispersion of the suspension.^[20]^ A visual assessment of mixedness can also done by observing the distribution of particles after mixing, as can be seen in Fig. 5(a), which shows a dense paste after 1 h of settling, where no major separation has occurred (white above the red line is residue on the vial wall) and in Fig. 5(b) at approximately 23 h, where the large particles settle to the bottom (below the blue line) and the small localize on the top (between the red and blue lines), a phenomenon commonly called “creaming.” Dyes and colored particles have also been used to show a more clear visual difference^[81]^ Overall, assessing homogeneity of a dense paste of particles, especially when multiple particle types are included, is a challenge. However, the large size of the macroscale particles improves the quality of optical methods, which are usually faster and less expensive than those needed for nanoscale particles, enabling more measurements and collection of representative statistics.

### Printing

**Figure 6:**
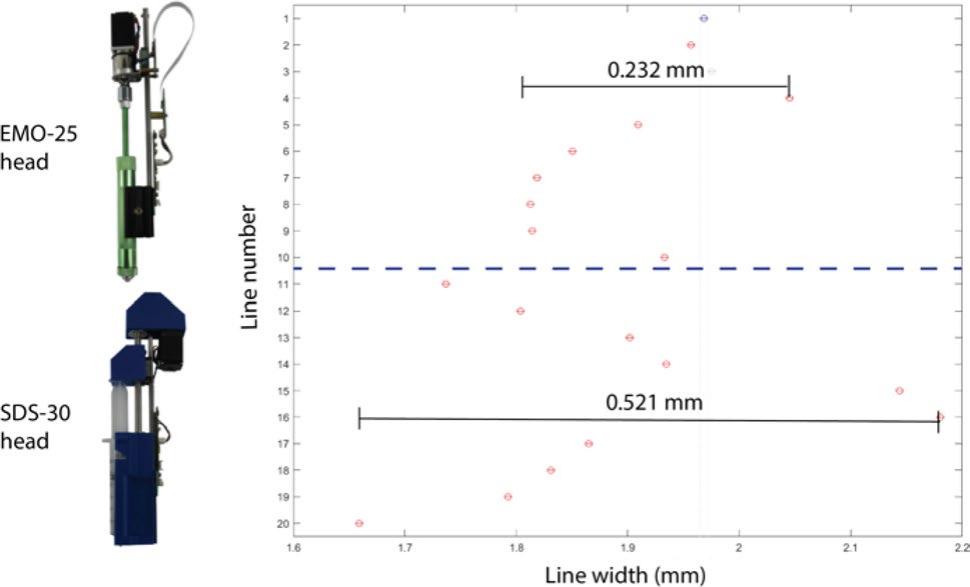
Line widths of play-doh printed using a Hyrel Hydra 3D printer with an EMO-25 print head (lines 1–10) and an SDS-30 print head (lines 11–20). Line width range was 0.232 mm with the EMO print head and 0.521 mm with the SDS-30 print head.

Challenges to processing dense pastes containing macroparticles also occur during the printing extrusion process. The high solid content in the formulation and the sudden constriction leading to the nozzle entrance in the syringe barrel frequently cause flow complications. Due to their high viscosities, dense pastes require high applied pressures to initiate and sustain steady-state extrusion. Defects due to high pressure demands can be minimized with the use of appropriate print heads: conical or tapered nozzles rather than straight nozzles, along with appropriate length/diameter ratios of the nozzle.^[82]^ Conical or tapered nozzles reduce the pressure demands on the extruder and allow dense suspensions to reach steady-state flow more quickly than straight-walled nozzles.^[82]^ Figure 6 compares the width of lines printed with modular heads sold by Hyrel 3D, showing that the variability in line width is much higher for the print head with the plastic syringe (SDS-30), which has a smaller nozzle and larger L/D ratio than the head with the metal syringe (EMO-25), highlighting the need for a robust print head and nozzle selection. For macroparticles, the chosen nozzle size should generally be at least ten times larger than the widest particle or agglomerate diameter at the exit to accommodate the flow of the suspension, otherwise phase separation and binder filtration can occur.^[83,84]^ Despite this, most readily available conical nozzles are smaller than 1.6 mm in diameter and are therefore unsuitable for use with particles larger than 200 µm. A nozzle gauge or diameter at the exit that is too small will produce a particle mat, and cause a blockage from the barrel constriction to the nozzle exit, resulting in failed extrusion.^[85]^ Incompatible nozzle sizes are usually qualitatively identified, either through observed discontinuous extrusion, binder leaching, or increased extrusion force required to push out material. The formation of a particle mat due to incompatible nozzle geometry is problematic for dense pastes since it can render unusable a portion of the suspension, resulting in wasted material. Furthermore, printed parts are then produced without control over the particle and binder distribution throughout the print.

**Figure 7:**
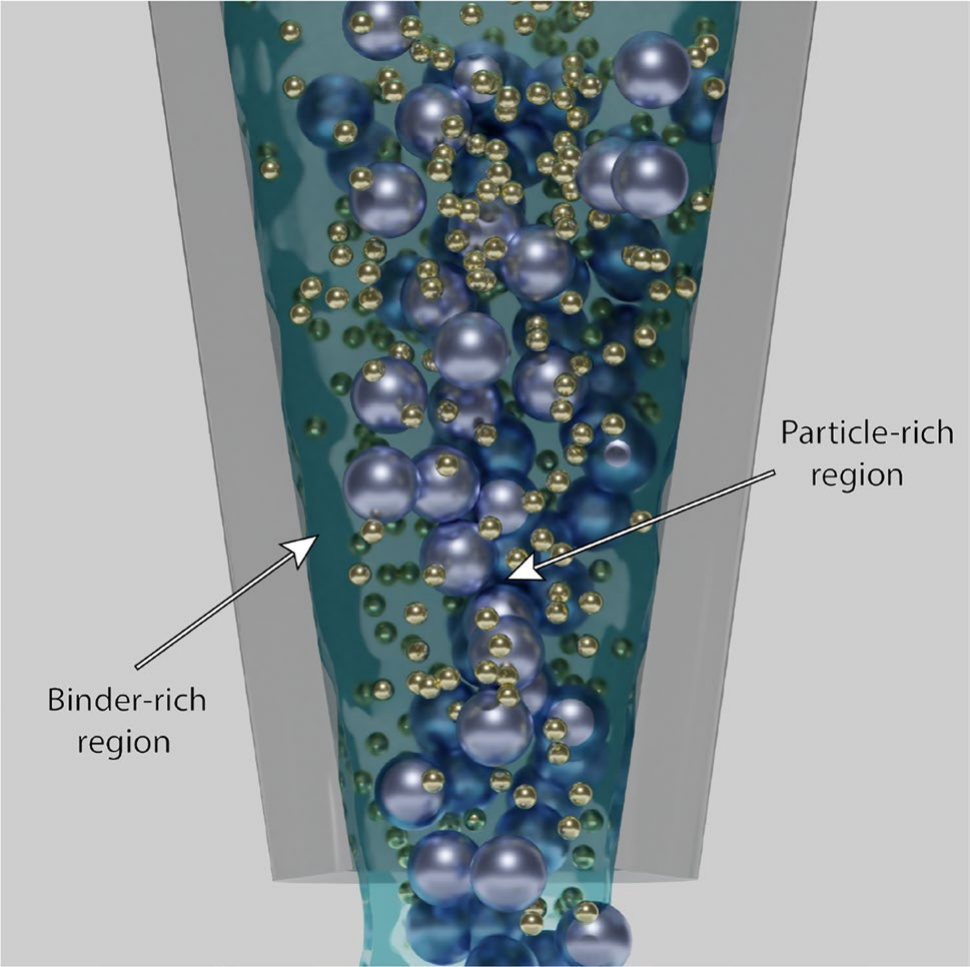
Visual representation of the slip layer and phase separation inside the printing nozzle.

The differences in flow characteristics between the binder and the particles in dense pastes present an additional consideration for 3D printing of these materials. As a result of the pressure drop applied across the extrusion nozzle, a binder-rich slip layer can form at the nozzle wall and further affect the spatial distribution of solid particles within the extruded paste (Fig. 7). Suspensions of dense pastes can phase separate within the nozzle; this occurs as the binder gradually leaches to near the wall of the nozzle where the shear stresses are highest.^[86–88]^ The slip layer in the syringe nozzle is directly affected by the nozzle geometry, where reducing the nozzle length or increasing the nozzle diameter contributes to preventing the formation of a slip layer significant enough to cause phase separation in the suspension.^[86,89]^ When dense pastes of colloidal or smaller particle sizes are considered, the formation of the slip layer becomes less common and extrusion problems are reduced. This is due to the smaller interstitial space between particles of smaller (< 10 μm) diameters preventing binder filtration.^[89]^ However, the higher viscosity of pastes composed of colloidal particles requires greater applied pressures to initiate and sustain extrusion compared to particles of larger diameters.^[90]^ Tailoring the suspension formulation or the printer’s hardware may therefore be necessary in order to achieve continuous extrusion of spatially homogenous dense pastes.

The last challenge we discuss regarding 3D printing of dense pastes is the post-extrusion suspension shaping. One of the major components that contributes to the shape of the extruded filament is the nozzle cross-sectional shape, but the height of the nozzle relative to the printer bed also contributes a significant effect. In general, to achieve a cylindrical filament, the extrusion flow rate must be sufficient enough to fill the spanning space dictated by the print layer height.^[91]^ However, shortening the distance between the nozzle exit and the print substrate can squeeze the extruded filament and change the extruded line width as well as create a top surface that is less rounded.^[11,82]^ This latter effect is beneficial to promote inter-layer adhesion, providing a flatter surface on which to extrude the subsequent layer.^[82,92]^ The increase in line width is also beneficial for reducing air voids between adjacent filaments.^[11,93]^ However, the bulging of the ink at the sides of the nozzle that results from a shortened print layer height can be disadvantageous to shape fidelity, and parts may require post-processing to smooth out outer walls.^[94]^ The authors also note that a reduction of the print layer height may cause additional suspension jamming and binder filtration, especially when the layer height nears the diameter of large particles. Therefore, while the effects of print parameters on the 3D printing of dense pastes can be somewhat generalized as described above, several printer hardware choices and the achievability of dimensional limits are still heavily dependent on suspension formulation.

## Outlook

Printing dense pastes using direct ink write additive manufacturing has significant promise for developing high-performance and customized products for a number of industries, including energetic materials and pharmaceuticals, while also having the potential to improve resource utilization, whether of extraterrestrial regolith or of in-space waste products. Pushing the particle content to high solids leads to particular challenges in formulation and printing and unique considerations in development of the inks, while using macroscale particles provides some similarities to existing approaches with colloidal particles, but also some new areas of focus. Crucially, significant work remains to fully understand the impact of the formulation components on the printability and enable rational design of dense pastes for 3D printing.

First, managing the particle characteristics including size distribution, shape, surface chemistry, and roughness requires adopting approaches for particle engineering from other technologies, such as pharmaceutical wet granulation, a process of mixing drug and excipient particles.^[60]^ With many important macroscopic particles coming from waste or natural materials, there are inherent heterogeneities and non-spherical particles, so further fundamental studies on how these characteristics impact flow and printing, in addition to discovery of potential additives to mitigate challenges, will be necessary for broad adoption. We discussed a few specific areas of focus, including sieving to separate particle sizes, in situ particle size measurements, and particle engineering to produce spherical particles, but more work is needed to understand the limits of these techniques and how the thin layer of binder present in dense pastes changes the optimum conditions and behaviors from prior work with powder flow.

For any suspension, stability, in particular with respect to maintaining homogeneity throughout the suspension, is a key focus of research and development. In working with dense pastes of macroparticles, there are two crucial processes that must be overcome to maintain a homogeneous suspension: (1) settling and (2) binder leakage. Macroparticles settle under gravity more readily than colloidal particles and, though the hindered settling of dense pastes decreases this effect, there is still mobility for the particles due to the fluid binder. Understanding the rate and final state of the settling is crucial to a robust design and designing formulation elements, such as additives and controlling particle size distributions accordingly, will be necessary for further adoption of additive manufacturing using dense pastes. Additionally, further study is needed to determine how the material transfer (i.e., loading of the syringe), aging, and printing processes impact the homogeneity of dense pastes of macroparticles and how this depends on the storage conditions, printer hardware, pressures, etc.

 Finally, the specific processing challenges in working with dense pastes of macroparticles, pertaining to both mixing and printing, still require significant fundamental studies and optimization. Two mixing methods have emerged as leaders, dual-axis centrifugal mixing and resonant acoustic mixing, but neither is universally optimal and further work is needed to link the formulation needs to the mixing process and necessary processing parameters. For 3D printing, the high solids content requires greater pressures and careful equipment design to ensure the highly viscous materials can be printed into desired shapes without leading to binder leakage and aggregation of the particles. Some approaches include tapered, wide nozzles and a balance of layer height and extrusion rate that leads to filament squeezing; however, ensuring versatility in printing dense pastes requires further analysis of the impacts of formulation and processing parameters on the quality of the printed parts. This versatility is very important in DIW of dense pastes because of the potential applications for using heterogeneous waste or extraterrestrial substitutes to Earth-native particulate materials, as well as for taking advantage of the compatibility of the DIW hardware with customized formulations. Thus, it is of particular importance to build off of the significant knowledge base discussed in this prospective to provide a full picture of formulation and processing factors and their impact on the quality of 3D printed parts from dense pastes of macroparticles.

## Data Availability

The datasets discussed in this study are available from the corresponding author on reasonable request.
